# Supplementary material for: Plant-Derived UDP-Glycosyltransferases for Glycosylation-Mediated Detoxification of Deoxynivalenol: Enzyme Discovery, Characterization, and In Vivo Resistance Assessment
Source: Toxins (Basel). 2025 Mar 22;17(4):153. doi: 10.3390/toxins17040153 (PMC12031568; doi:10.3390/toxins17040153)
Supplement: Supplementary file 1 [file toxins-17-00153-s001.zip › toxins-3510832-supplementary.pdf]

Supplementary Material: Plant-Derived UDP-Glycosyltransferases for Glycosylation-Mediated Detoxification of Deoxynivalenol: Enzyme Discovery, Characterization, and In Vivo Resistance Assessment

Table S1. Known plant UGTs active on DON, identified and partially characterized in the literature. n.a.: not available.

Enzyme	Organism	Accession (NCBI)	Reference
*At*UGT73C5	*Arabidopsis thaliana*	NP_181218	[31]
*Hv*UGT13248	*Hordeum vulgare*	ADC92550	[32]
*Bd*UGT74J9	*Brachypodium distachyon*	PNT60691	[36]
*Ta*UGT6	*Triticum aestivum*	XP_037443793	[33]
*Os*UGT9	*Oryza sativa*	XP_015635481	[34]
*As*UGT1	*Avena sativa*	n.a.	[37]
*As*UGT2	*Avena sativa*	n.a.	[37]

Table S2. LC-MRM/MS source parameters for the identification of DON and DON-3-Glc used in the GLY-it library screening.

Source parameter	Value
Ion Source	Electrospray Positive Mode (ESI+) and Electrospray Negative Mode (ESI–)
Capillary Voltage	3.5 kV
Nozzle Voltage	500 V
Source Gas Temperature	340°C
Source Gas Flow	12 L/min
Source Sheath Gas Temperature	380°C
Source Sheath Gas Flow	12 L/min
Nebulizer	30 psi
Mode	MRM

Table S3. LC-MRM/MS settings for the identification of DON and DON-3-Glc used in the GLY-it library screening.

Compound	RT (min)	ESI	Ionisation	Precursor ion (m/z)	Product ions (m/z)	Frag. Vol.	CE
DON	3.5	Pos	$[M+H]^+$	297	249	110	0
					231	110	0
					203	110	5
DON-3-Glc	3.68	Neg	$[M+HCOO]^-$	503	457	110	5
					427	110	10
					247	110	20

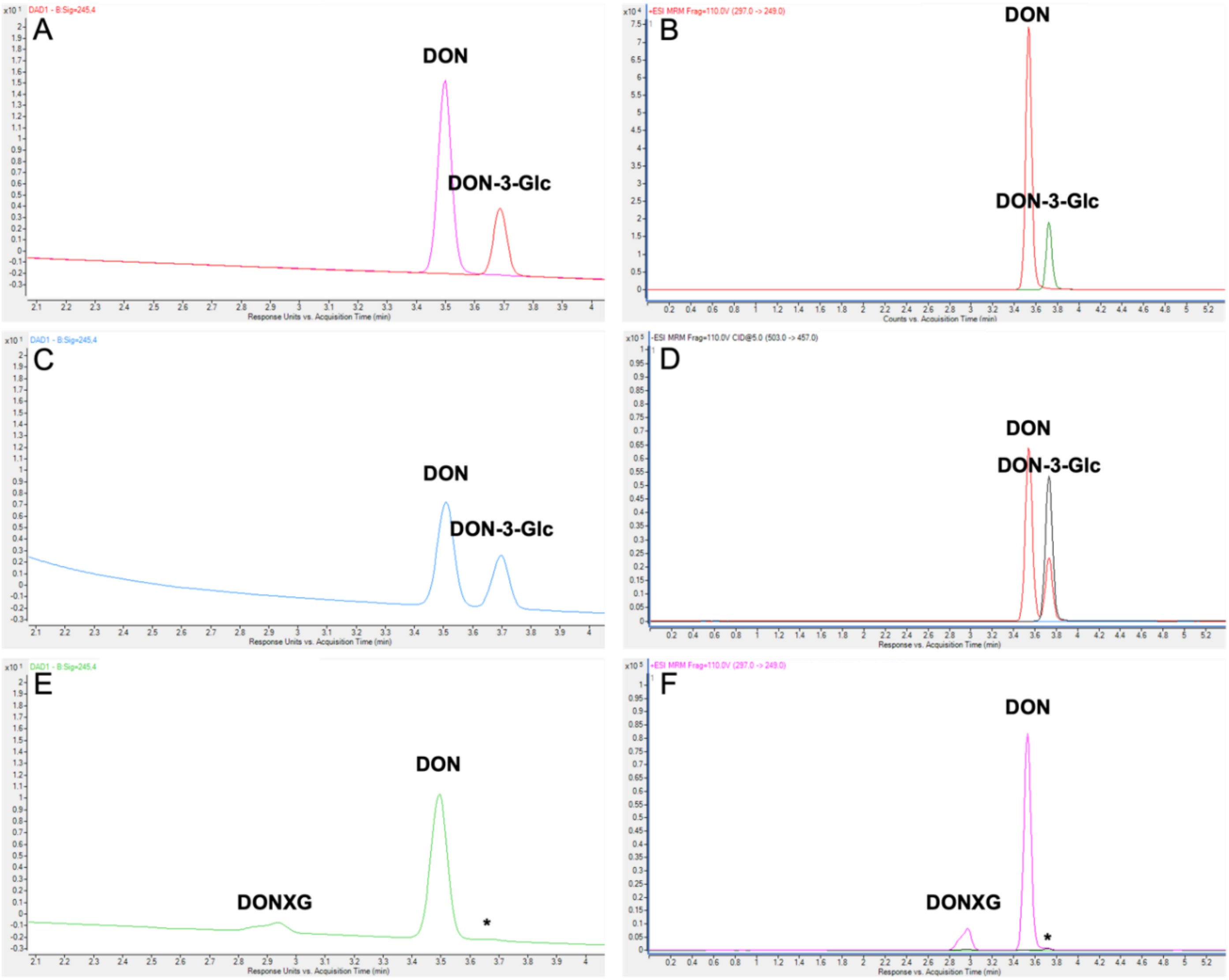

Figure S1. Selected UV and MS/MS chromatograms of analytical standards and enzymatic reactions of UGTs with DON analyzed by UHPLC-QqQ-MS from the GLY-it library screening. **(A)** Overlay of UV chromatograms for DON (in magenta) and DON-3-Glc (in red) standards. **(B)** Overlay of MS/MS chromatograms for DON (in red, *m*/*z* 297 > *m*/*z* 249) and DON-3-Glc (in green, *m*/*z* 503 > *m*/*z* 457) standards. **(C)** UV chromatogram showing the enzymatic reaction of *Zj*UGT producing DON and DON-3-Glc. **(D)** MS/MS chromatogram showing the *m*/*z* 297 > *m*/*z* 249 transition (in red) and the *m*/*z* 503 > *m*/*z* 457 transition (in black) from the enzymatic reaction of *Zj*UGT. **(E)** UV chromatogram showing the enzymatic reaction of *Bv*UGT, producing an unknown product referred to as DON-X-G (DONXG), DON, and traces of DON-3-Glc (*). **(F)** MS/MS chromatogram overlay showing the *m*/*z* 297 > *m*/*z* 249 transition (in magenta) and the *m*/*z* 503 > *m*/*z* 457 transition (in black) from the enzymatic reaction of *Bv*UGT, with traces of DON-3-Glc (*). Retention times are 2.95 min for DON-X-G, 3.50 min for DON, and 3.70 min for DON-3-Glc, with DON-3-Glc eluting after DON, likely due to the mobile phase composition used in the chromatographic separation.

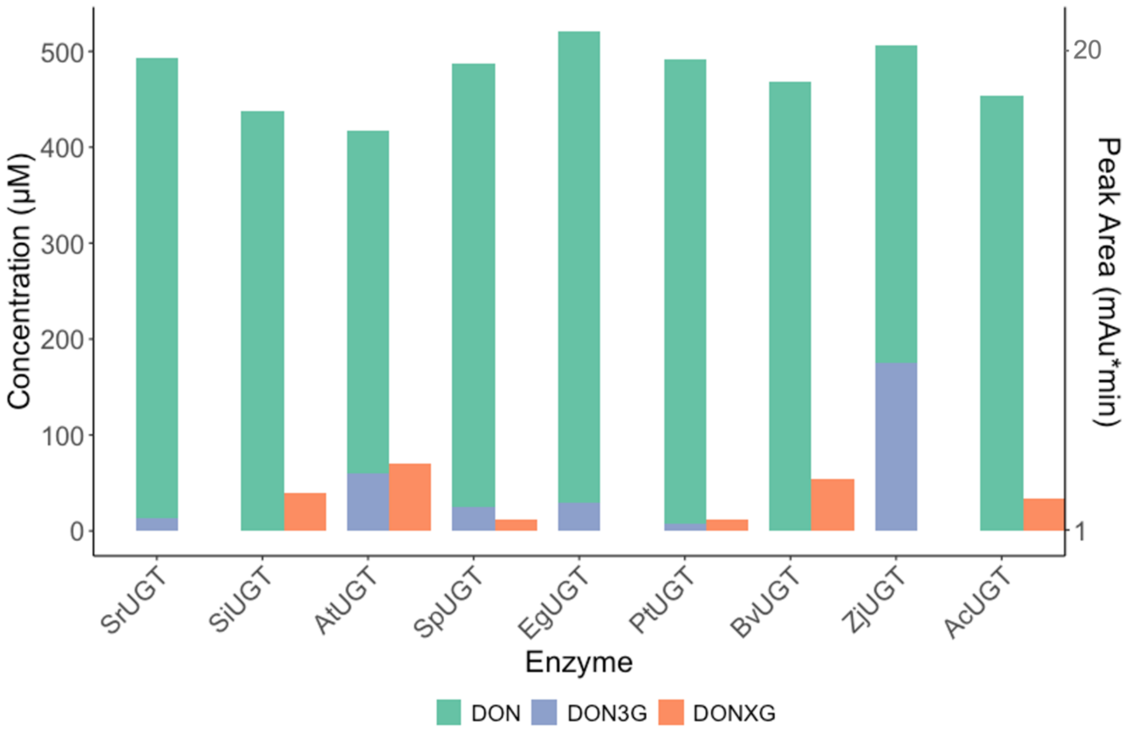

Figure S2. Bar plot showing the complete results of the 24-hour reaction from the GLY-it library screening with DON. The plot highlights the enzymes from the GLY-it library that exhibited activity on DON (µM), resulting in the formation of DON-3-Glc (DON3G, µM) and a secondary product tentatively identified as DON-X-G (DONXG, mAU*min). The screening results also identified *At*UGT73C5 (*At*UGT), previously reported in the literature [1]. Assays, carried out as single experiments, were performed using 5 µL of purified enzyme, 0.5 mM DON, and 1.25 mM UDP-Glc in a buffer containing 100 mM Tris-HCl, 5 mM $MgCl_2$, and 1 mM KCl (pH 7.4), supplemented with 0.2 U FastAP thermosensitive alkaline phosphatase. Reactions were quenched after 24 hours and analyzed by LC-MS/MS.

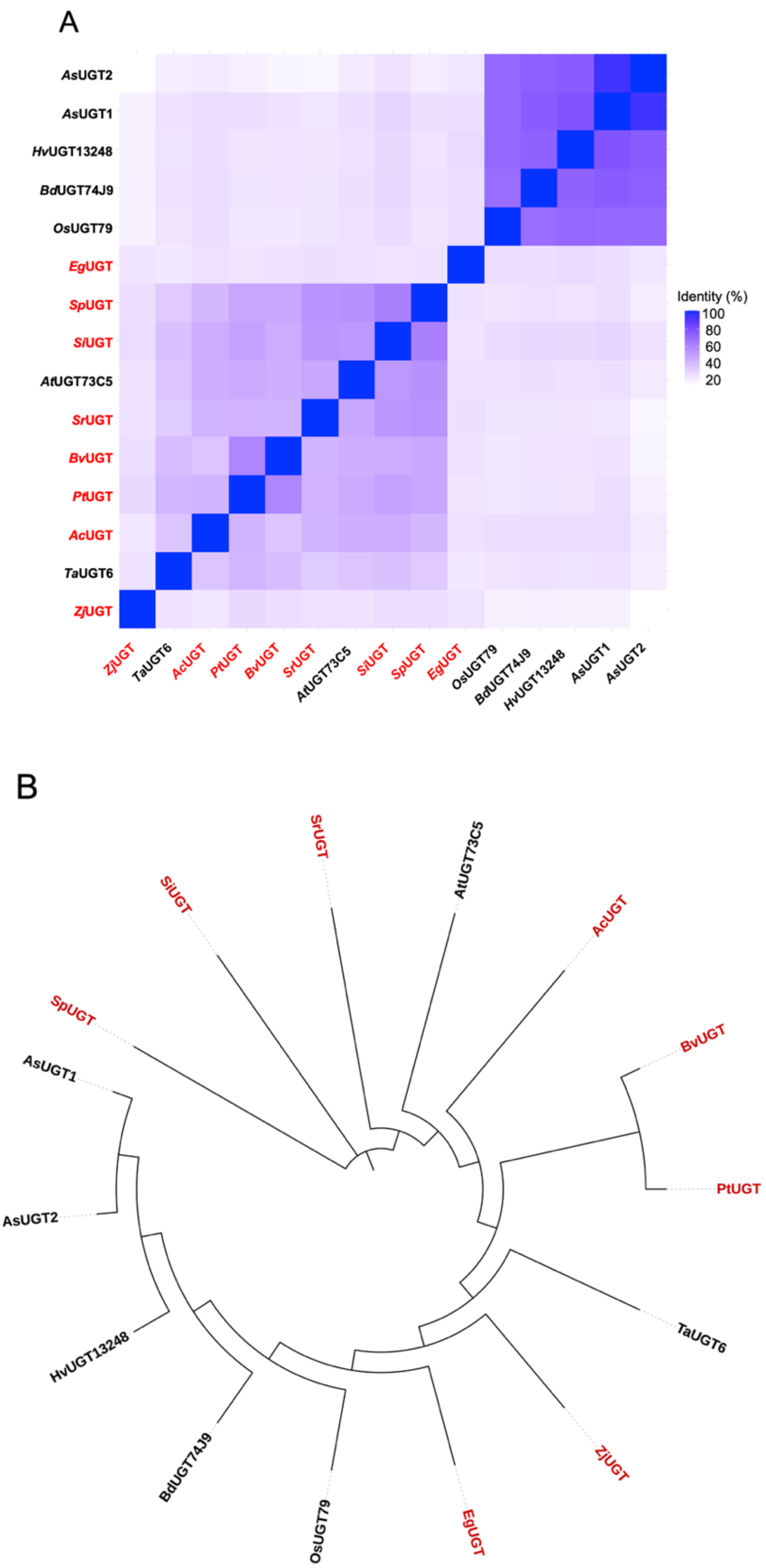

Figure S3. Identity matrix and phylogenetic tree of the reported DON UGTs from the literature (in black) compared to those identified in our study from the GLY-it library (in red). **(A)** Percentage identity matrix of previously known DON UGTs compared to those identified in our study. Multiple sequence alignment was performed using Clustal O, and the heat map was visualized in Rstudio. **(B)** Phylogenetic analysis comparing UGTs reported in the literature with those identified in our study. The phylogenetic tree was constructed using the neighbor-joining method in Clustal O and visualized in iTOL with default settings.

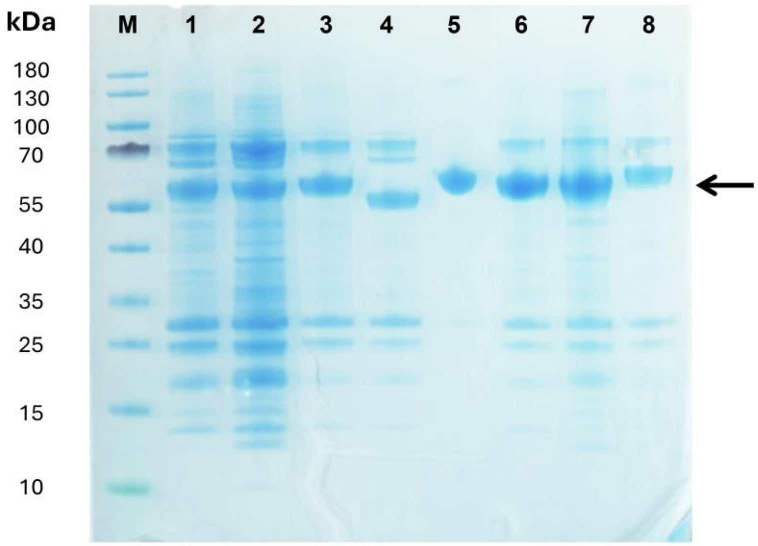

Figure S4. SDS-PAGE analysis of purified GLY-it UGTs with activity on DON after HisTrap™ purification. The black arrow indicates the UGTs, which have an approximate molecular weight of 55-60 kDa. M: PageRuler Prestained Protein Ladder (Thermo Fisher Scientific); 1: *Sr*UGT; 2: *Si*UGT; 3: *Sp*UGT; 4: *Eg*UGT; 5: *Pt*UGT; 6: *Bv*UGT; 7: *Zj*UGT; 8: *Ac*UGT.

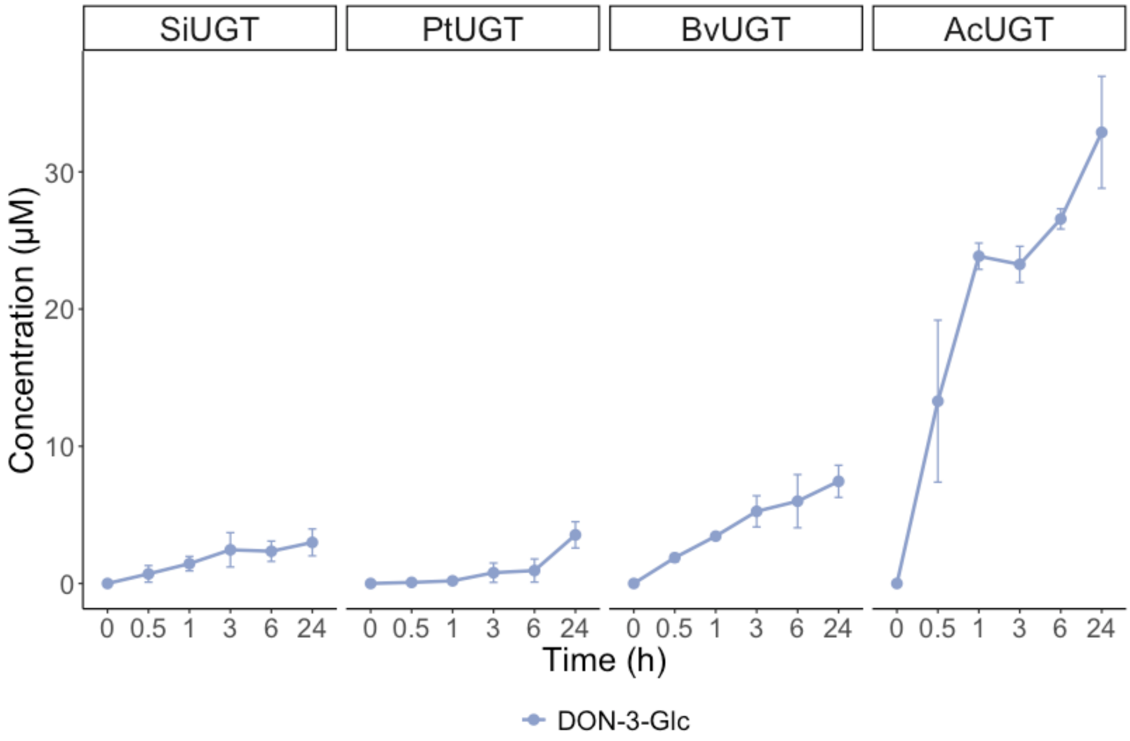

Figure S5. Zoomed-in time-course analysis of the glycosylation reaction of DON (0-24 hours) catalyzed by the recombinant *Si*UGT, *Pt*UGT, *Bv*UGT, and *Ac*UGT, resulting in the formation of DON-3-Glc. Error bars indicate the standard deviation from the mean of duplicate measurements. Each assay was conducted with 0.75 mg/mL of purified enzyme, 0.5 mM DON, and 2 mM UDP-Glc in a 50 mM sodium-phosphate buffer at pH 7.5. Reactions were quenched at specific time intervals and analyzed by reverse-phase HPLC.

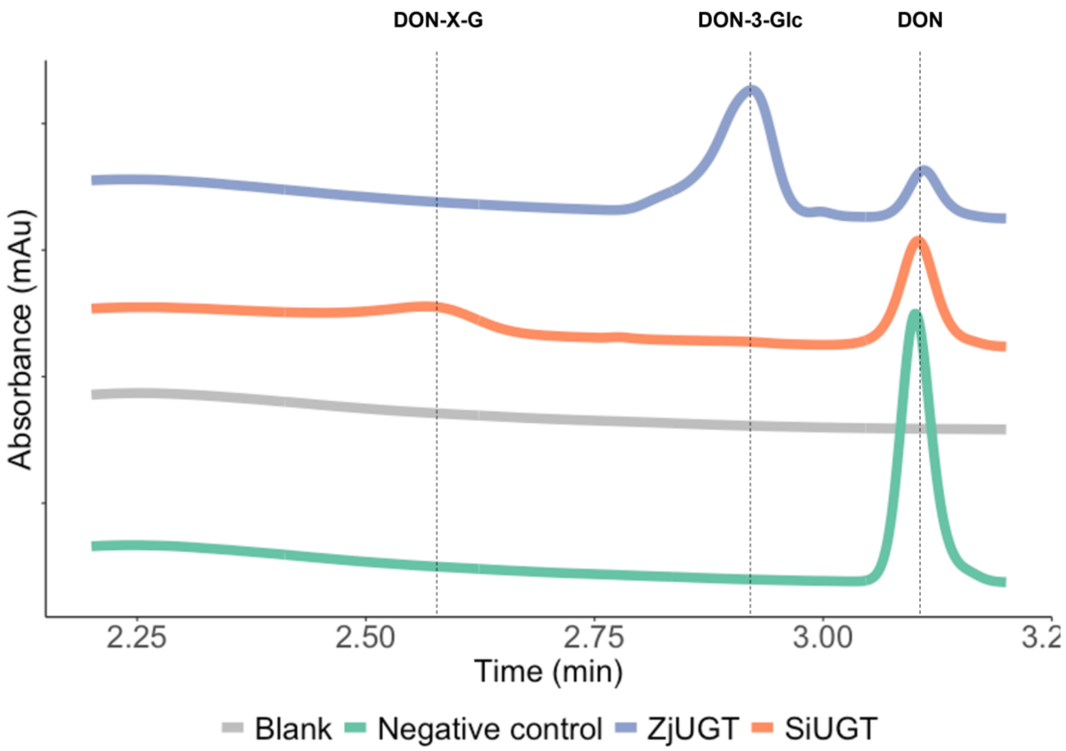

Figure S6. Example of reverse-phase HPLC chromatogram of the enzymatic reactions of UGTs with DON. The reactions with *Si*UGT and *Zj*UGT are displayed in orange and light blue, respectively. A control reaction without the enzyme is shown in green, while a blank sample is indicated in grey. The retention times are as follows: 2.60 min for DON-X-G, 2.90 min for DON-3-Glc, and 3.10 min for DON.

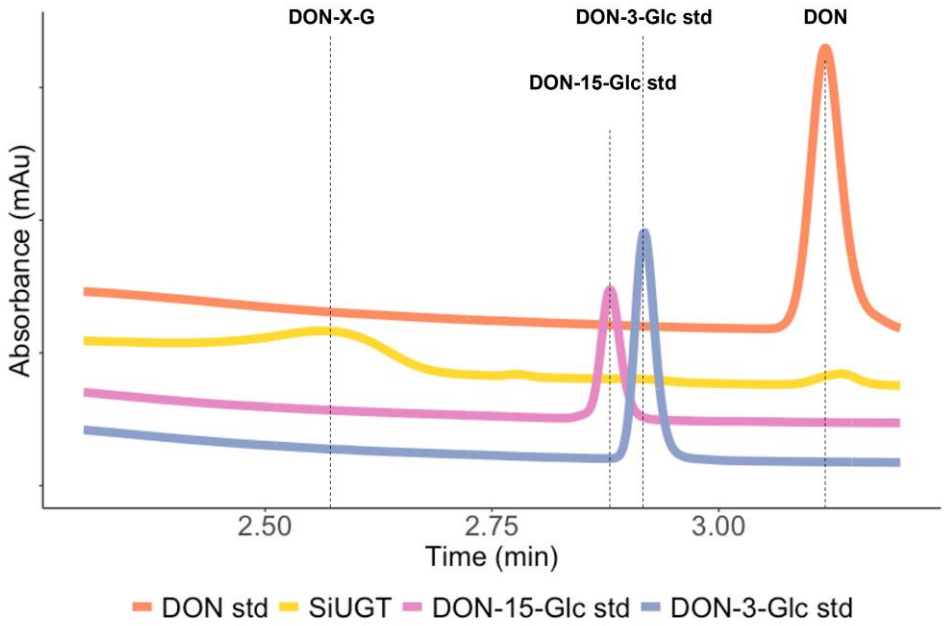

Figure S7. Reverse-phase HPLC chromatogram comparing the enzymatic reaction of *Si*UGT with DON to analytical standards for DON-15-Glc, DON-3-Glc, and DON. The enzymatic reaction of *Si*UGT is shown in yellow, while the analytical standards (std) are represented in pink (DON-15-Glc), light blue (DON-3-Glc), and orange (DON). The retention times are as follows: 2.60 min for DON-X-G, 2.85 min for DON-15-Glc, 2.90 min for DON-3-Glc, and 3.10 min for DON.

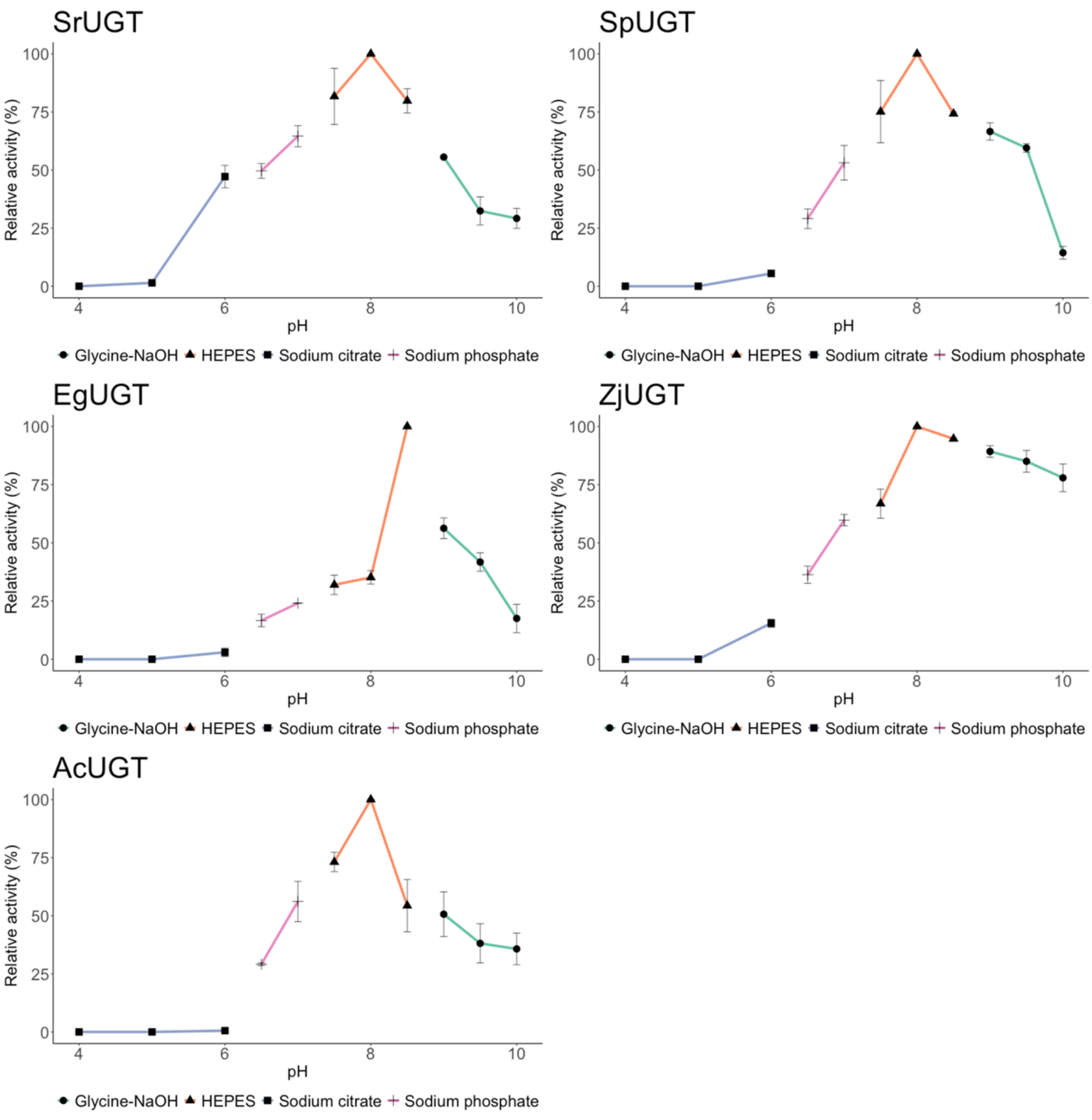

Figure S8. pH activity profiles of the identified UGTs. The effect of pH on the activity of the top DON-3-Glc producing UGTs identified in the GLY-it library screening. Enzymes (0.15 mg/mL) were assayed with 0.25 mM DON and 1 mM UDP-Glc at 22 °C using a range of buffers: 50 mM sodium citrate (pH 4, 5, 6), sodium phosphate (pH 6.5, 7), HEPES (pH 7.5, 8, 8.5), and glycine (pH 9, 9.5, 10). Error bars indicate the standard deviation from the mean of duplicate measurements.

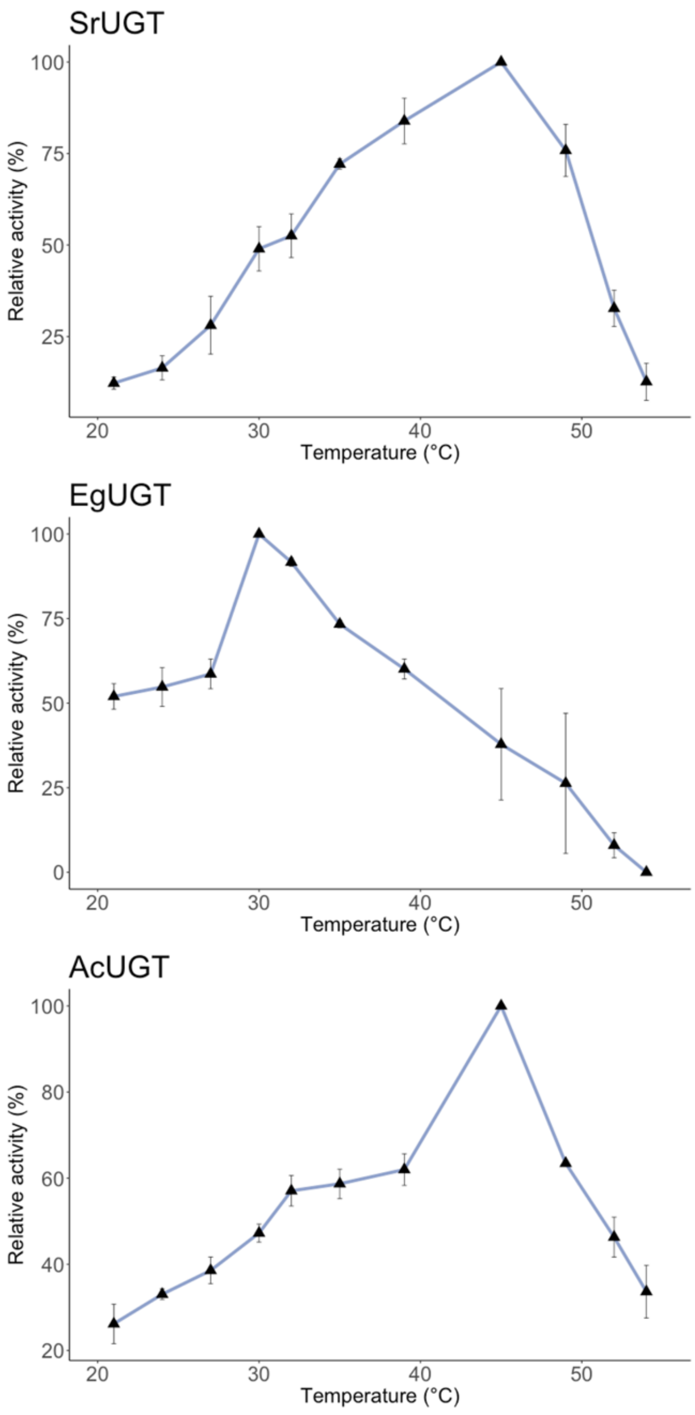

Figure S9. Temperature activity profiles of the identified UGTs. The effect of temperature on the activity of the top DON-3-Glc producing UGTs identified in the GLY-it library screening. Enzymes (0.15 mg/mL) were assayed with 0.25 mM DON and 1 mM UDP-Glc at 22 °C in a 50 mM sodium phosphate buffer, pH 7.5, across a temperature range of 20-55 °C. Error bars indicate the standard deviation from the mean of duplicate measurements.

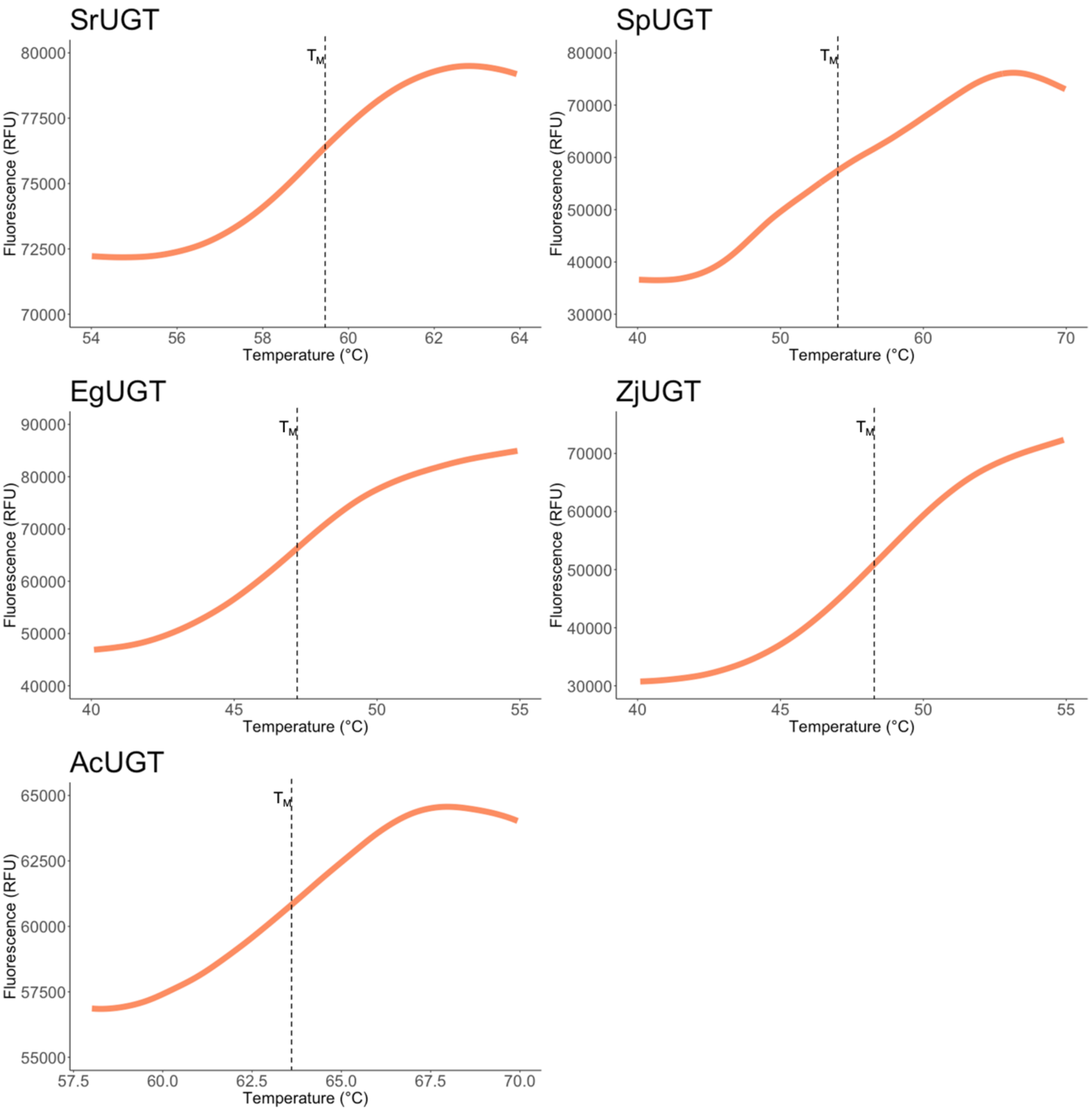

Figure S10. Thermostability profiles of the identified UGTs. The effect of temperature on the thermostability of the top DON-3-Glc producing UGTs identified in the GLY-it library screening, assessed through differential scanning fluorimetry (DSF). Each UGT was tested at a concentration of 0.8 mg/mL in a 50 mM sodium phosphate buffer (pH 7.5). The data shown represent the mean values from three independent experiments.

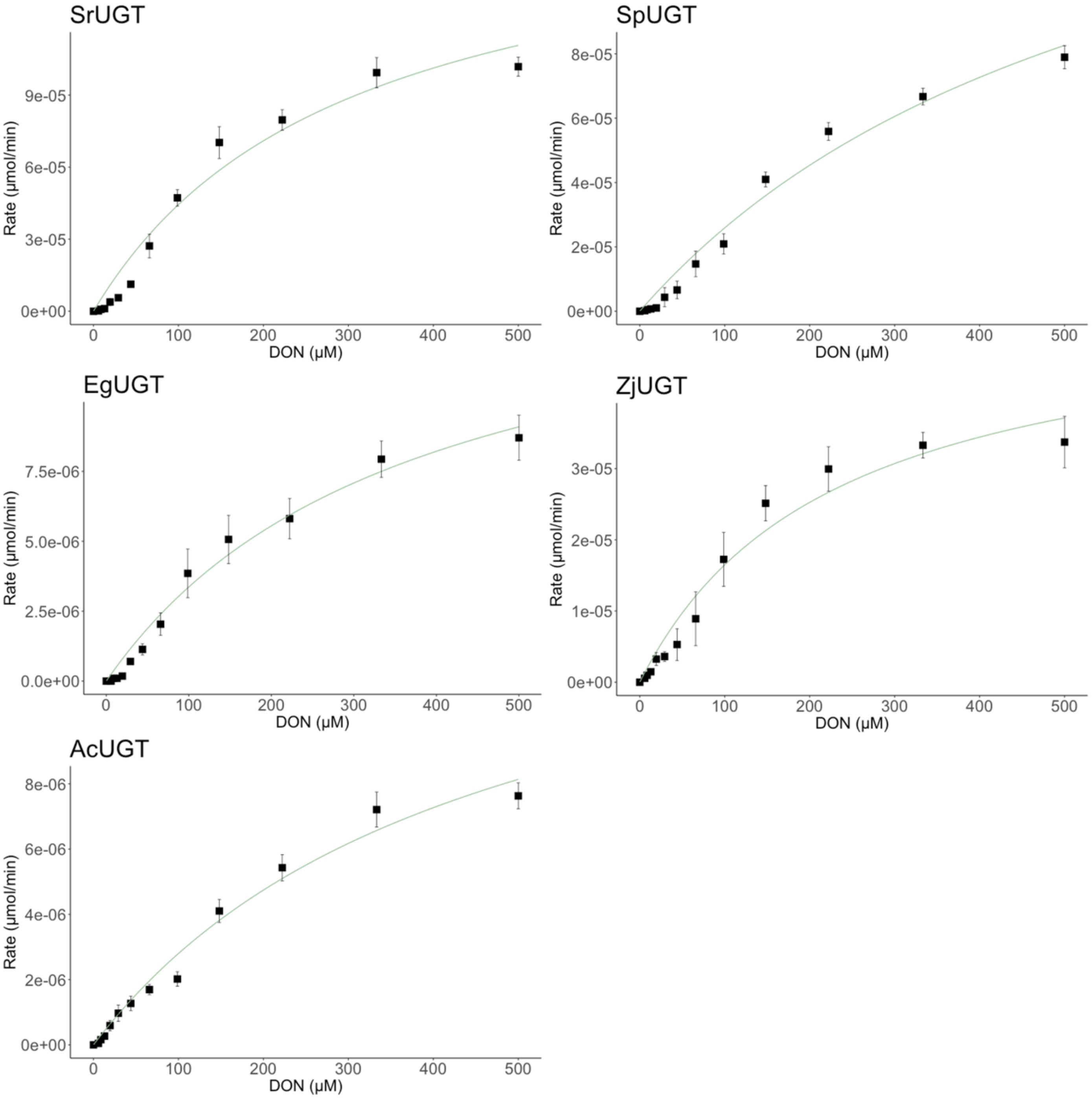

Figure S11. Kinetic characterization of the identified UGTs. Michaelis-Menten curves illustrating the enzymatic activity of the top DON-3-Glc producing UGTs identified from the GLY-it library screening. Each reaction was conducted at the optimal temperature and pH for each enzyme, using 0.1-0.6 mg/mL UGT, and monitored over 15 minutes. Error bars indicate the standard deviation from the mean of duplicate measurements. Kinetic parameters were determined by fitting the data to the Michaelis-Menten model using the *drc* package in Rstudio.

Table S4. List of primers used in this study for amplifying UGT genes from GLY-it plasmids for subsequent cloning into the *S. cerevisiae* vector. Uppercase letters correspond to sequences that anneal to the target DNA, while lowercase letters represent primer extensions. Restriction sites for HindIII and NotI are highlighted in bold.

Enzyme	Primer name	Sequence (5′-3′)
SrUGT	SrUGT_F	ttcagaagaggacttatca**aagctt**ATGAGCGGTAGCATGGCAC
	SrUGT_R	tcgagctccaccgcggtg**gcggccgc**TTAGCGGTTTTTGCACAGC
SiUGT	SiUGT_F	aatc**aagctt**ATGGCCATTCGTGAACAAGAACCG
	SiUGT_R	actc**gcggccgc**TTATTTTTCTTGCTGCAGACCACCG
SpUGT	SpUGT_F	TTAATTTCAGAAGAGGACTTATCA**aagctt**ATGGGTGTTCTGACCATTGAAC
	SpUGT_R	GAATTCGAGCTCCACCGCGGTG**gcggccgc**TTACATCGGTTTCAGAATTTTTGC
EgUGT	EgUGT_F	aatc**aagctt**ATGGAAAATCAGGGTCGTAATG
	EgUGT_R	tgcc**gcggccgc**TTACAGGCTGGTCAGCAGTTC
PtUGT	PtUGT_F	aatc**aagctt**ATGGGTAGCCTGGGTCATC
	PtUGT_R	actt**gcggccgc**TTACGGACGACGCCAGCG
BvUGT	BvUGT_F	ATTTCAGAAGAGGACTTATCA**aagctt**ATGGGTGCAGAACCGCAGCGTC
	BvUGT_R	GAATTCGAGCTCCACCGCGGTG**gcggccgc**TTACAGATACTGTTTATAGCCATTGG
ZjUGT	ZjUGT_F	ttcagaagaggacttatca**aagctt**ATGAAAAAAGCCGAACTG
	ZjUGT_R	tcgagctccaccgcggtg**gcggccgc**TTAGCTCACGTTTTCCAG
AcUGT	AcUGT_F	actg**aagctt**ATGAAAGATGTGACACCGC
	AcUGT_R	atct**gcggccgc**TTAGGTAACAACAACATGTGC

Disclaimer/Publisher's Note: The statements, opinions and data contained in all publications are solely those of the individual author(s) and contributor(s) and not of MDPI and/or the editor(s). MDPI and/or the editor(s) disclaim responsibility for any injury to people or property resulting from any ideas, methods, instructions or products referred to in the content.
